# Deciphering between enhanced light emission and absorption in multi-mode porphyrin cavity polariton samples

**DOI:** 10.1515/nanoph-2023-0748

**Published:** 2024-03-15

**Authors:** Elizabeth O. Odewale, Aleksandr G. Avramenko, Aaron S. Rury

**Affiliations:** Materials Structural Dynamics Laboratory, Department of Chemistry, 2954 Wayne State University, 48202, Detroit, MI, USA

**Keywords:** strong light–matter coupling, cavity quantum electrodynamics, cavity-enhanced light–matter interactions

## Abstract

It remains unclear how the collective strong coupling of cavity-confined photons to the electronic transitions of molecular chromophore leverages the distinct properties of the polaritonic constituents for future technologies. In this study, we design, fabricate, and characterize multiple types of Fabry-Pérot (FP) mirco-resonators containing copper(II) tetraphenyl porphyrin (CuTPP) to show how cavity polariton formation affects radiative relaxation processes in the presence of substantial non-Condon vibronic coupling between two of this molecule’s excited electronic states. Unlike the prototypical enhancement of Q state radiative relaxation of CuTPP in a FP resonator incapable of forming polaritons, we find the light emission processes in multimode cavity polariton samples become enhanced for cavity-exciton energy differences near those of vibrations known to mediate non-Condon vibronic coupling. We propose the value of this detuning is consistent with radiative relaxation of Herzberg-Teller polaritons into collective molecular states coupled to the cavity photon coherently. We contrast the feature stemming from light emission from the HT polariton state with those that occur due to polariton-enhanced light absorption. Our results demonstrate the landscape of molecular and photonic interactions enabled by cavity polariton formation using complex chromophores and how researchers can design resonators to leverage these interactions to characterize and control polaritonic properties.

## 1 Introduction

The strong coupling of cavity photonic fluctuations and the electronic transitions of an ensemble of molecules leads to the formation of collective light–matter states known as cavity exciton polaritons [1], [2], [3], [4], [5]. The polariton states formed through strong light–matter coupling possess both the coherent delocalization of cavity photons and the intrinsic coupling of electronic states present in localized molecules [6], [7], [8]. This combination of properties has enabled novel forms of energy transfer in polariton platforms [9], [10], [11], [12]. Furthermore, researchers propose that cavity polariton formation enables new handles over chemical reactivity due to delocalized nature of the cavity photons [3]. These results suggest that cavity polariton formation could provide coherent, delocalized material systems from which transformative technologies in light harvesting, energy transduction, and chemical synthesis could be harnessed.

Metalloporphyrins resemble organometallic molecules that are involved in both natural and artificial photosynthesis [13], [14], [15], [16], [17], [18], [19], [20], [21], [22], [23]. These molecules possess relatively complex electronic structures due to accidental degeneracy of two frontier molecular orbitals, *a*_1*u*_ and *a*_2*u*_, which are composed of electron density on distinct atoms of the porphyrin ring. The degeneracy of these orbitals leads to strong interactions which mixes them in excited state configurations and leads to the formation of two new spectroscopic states denoted *B* and *Q*, as detailed in the seminal work of Goutermann [24]. In the case of natural photosynthesis, plants and bacteria have found less symmetric ringed chromophores like porphyrins that enable efficient light absorption and energy transfer mechanisms. However, the relatively reduced symmetry of these natural chromophores and their aggregates complicates predictions of their optical and dynamical behavior using first principles methods. Based on this deficiency, researchers need methods to leverage the relatively more straightforward models explaining the photophysics of metalloporphyrins to design optical spectra and collective phenomena to control light harvesting, energy transfer, and photochemical behavior. Cavity exciton polariton formation may represent such a method if the researchers can understand how the fundamental photophysics of these molecular systems affects the hybrid light–matter states they form.

Recently, we used both theoretical and experimental approaches to motivate a physical picture in which the simultaneous vibrationally mediated intramolecular coupling of the metallporphyrin *B* and *Q* exciton states and strong light–matter coupling within a Fabry–Pérot (FP) micro-cavity leads to the presence of Herzberg–Teller (HT) polaritons [8]. Using photoluminescence spectra excited under non-resonant conditions, we proposed that the non-Condon coupling between the *B* and *Q* exciton states of copper(II) tetraphenyl porphyrin (CuTPP) mediated by a non-totally symmetric vibration enables a transfer of photonic content between high lying cavity polariton states to a dispersive state whose energy lies below those of localized molecular states. Based on theories of cavity polariton formation within the Condon approximation developed by Spano previously [25], [26], we hypothesized that the presence of a resonator mode near the energy of the proposed HT polariton state enhances the radiative relaxation. Despite the initial results that supported these proposals, the lack of resonant excitation of the cavity polariton states and minimal detection of the dispersive cavity emission in these samples limited our ability to characterize both the cavity and HT polaritons more completely.

**Figure 1:**
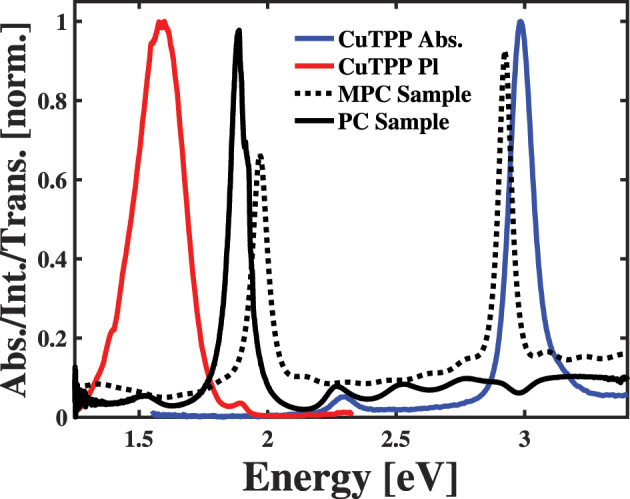
Comparison of the absorption (blue) and light emission (red) spectra of copper(II) tetraphenyl porphyrin to the transmission spectra of single layer (solid black) and multilayer (dashed black) Fabry–Pérot micro-cavities designed to enhance light absorption and emission processes.

In this study, we use precision fabricated FP micro-cavity samples and angularly resolved spectroscopy under resonant conditions to assess how the presence of cavity modes affects the radiative relaxation from HT polaritons. To enable these determinations, we fabricated two sets of FP micro-cavity samples, as explained in the Methods section of the Supporting Information (SI) document. In one sample, we fabricated distributed Bragg reflectors (DBRs) possessing single photonic stopbands near 1.9 eV, formed single CuTPP-doped polymer layers, and capped these structures with ∼15 nm Al layers. This process leads to the formation of a single, *λ*/2 cavity mode near 1.9 eV, which we show as the solid black transmission spectrum in the top panel of Figure 1. As seen in the comparison in the top panel of Figure 1, the peak in this transmission spectrum overlaps significantly with the low intensity feature of the CuTPP light emission spectrum, which corresponds to radiative relaxation of this molecule’s ^2^ *Q* exciton state. The doublet nature of this state results from the delocalization of the unpaired electron of the Cu^2+^ cation to the interior nitrogen atoms of the porphyrin macrocycle [27]. We propose that the overlap between the resonator and chromophore spectra will lead to weak light–matter coupling between the cavity photons and molecular excited states, which will manifest itself as an increased radiative relaxation sometimes referred to as the Purcell effect [28]. Based on this expectation, we refer to this set of structures as the Purcell Cavity (PC) samples. We show the structure of these samples schematically in Figure S3 of the Supporting Information document.

In the other set of samples, we use multimode distributed Bragg reflectors possessing 3*λ*/4 layers to form multiple photonic stopbands. We then formed an intracavity region composed of three layers in which we embed CuTPP in two non-adjacent layers, as described previously [8] and overviewed in the Supplementary Material document. When we cap this structure with an ∼15 nm film of Al, we form a structure possessing two high quality cavity modes, as shown in the dashed transmission spectrum in the top panel of Figure 1. When tuned to the appropriate angle, the higher energy of these two cavity modes moves into resonance with the intense Soret transition to the *B* exciton of CuTPP, as seen in the blue spectrum in the top panel of Figure 1, and drives polariton formation through strong light–matter coupling. At the same time, the lower energy of the two cavity modes lies close to that of the emission from the *Q* exciton of resonator-embedded CuTPP molecules, which is shown in red in the top panel of Figure 1. We refer to this set of structures as the Multilayer Polariton Cavity (MPC) samples and show a representative schematic of their structures in Figure S4 of the Supporting Information document. Our results and the proposed explanation for their appearance in our measurements helps researchers understand how to leverage porphyrin chromophores in intricate photonic structures to leverage their fundamental properties to control light emission spectra and properties.

## 2 Results

**Figure 2:**
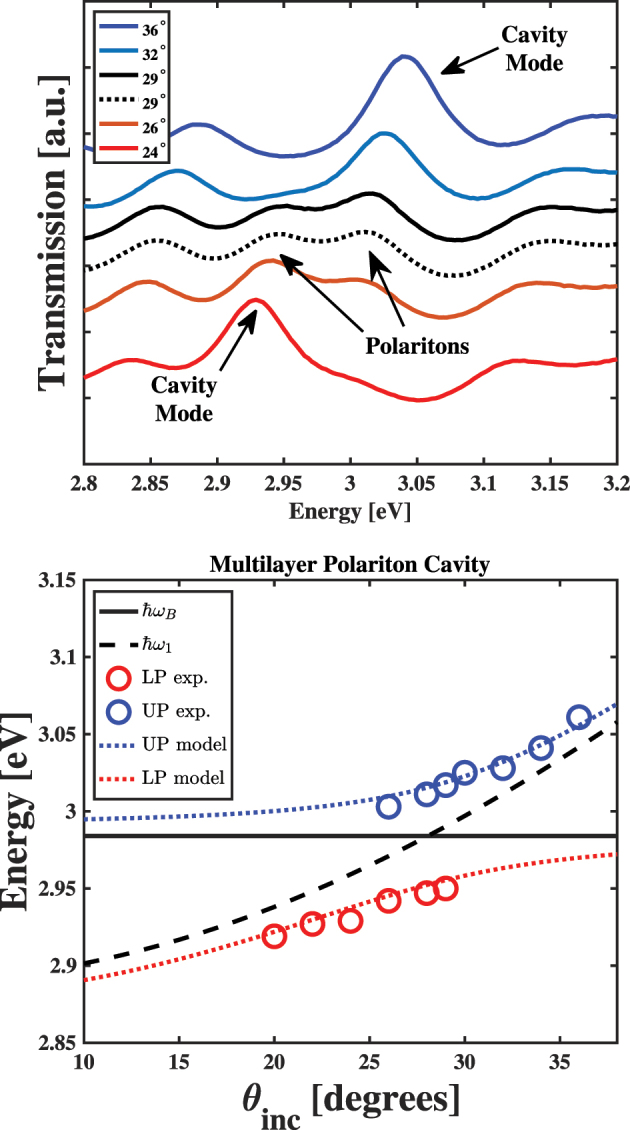
(Top panel) Angularly resolved transmission spectra of the multilayer polariton cavity sample showing the avoided crossing of the cavity photon mode and the CuTPP Soret transition resulting in the appearance of two distinct peaks at *θ*_inc_ = 29°. At angles below this value, we only observe the peak due to transmission through the cavity at energies below the Soret transition of CuTPP. In addition, at angles above 29°, we only observe the peak due to transmission through the cavity at energies above the Soret transition of CuTPP, as indicated in the figure. (Bottom panel) Comparisons between the peak energies of the UP (blue circles) and LP (red circles) found from experiment to energies of the UP (blue dotted line) and LP (red dotted line) produced by a matrix model. The energies of CuTPP *B* excitons and cavity photons used to produce model results are shown as solid black and dashed black lines, respectively.

The top panel of Figure 2 shows the experimental transmission spectra of the MPC sample for several different representative incidence angles in the region near the Soret transition of CuTPP. By fitting these spectra to Lorentzian functions and extracting their central energies, we can reproduce the expected anti-crossing of the transmission peaks, which is the spectral signature of cavity polariton formation [3]. We compare the experimental peak energies to models’ polariton states produced by diagonalizing a 2 × 2 Hamiltonian matrix explained previously [7], [8], which we show in the bottom panel of Figure 2. The good agreement between our experimental and model results suggests that the MPC sample supports cavity polaritons formed from CuTPP *B* excitons.

**Figure 3:**
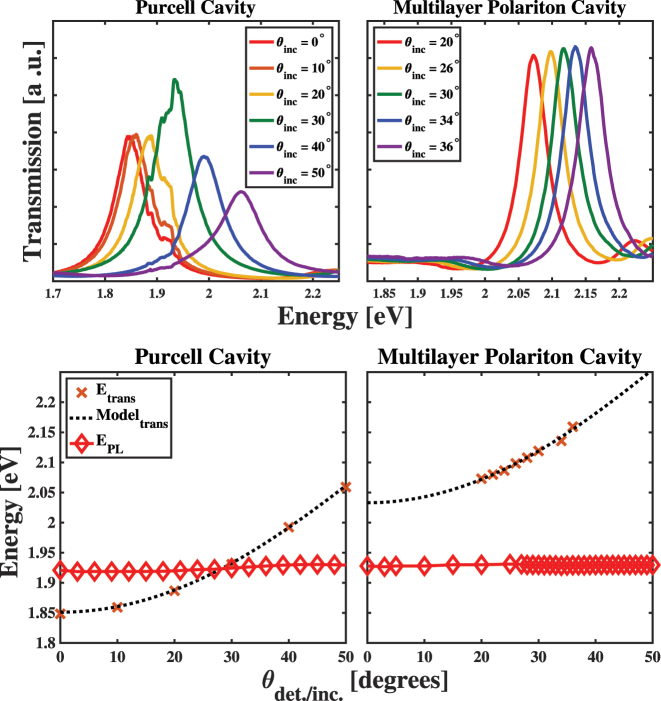
(Top panels) Dispersion of the resonator mode near the CuTPP *Q* state light emission in the Purcell cavity (left) and multilayer polariton cavity (right) samples as a function of a probe beam’s incidence angle onto each structure. (Bottom panels) Comparisons between the angle-dependent energies of the resonator modes (crosses) to those of light emission peaks (diamonds) in the Purcell cavity (left) and multilayer polariton cavity (right) samples.

To better understand the effects of cavity modes present in different spectral regions of the material’s optical response on the properties of our samples, we measured the angle-dependent properties of our samples’ optical spectra, which included measurements of both their transmission and light emission spectra. The top panels of Figure 3 show the transmission spectra of the PC and MPC samples in the spectra region near the light emission of the ^2^ *Q* exciton of CuTPP found using the methods described in the Supporting Information. The angle-dependent energies of the observed peaks demonstrate the dispersive nature of FP micro-resonator mode, which suggest the successful formation of well-defined cavity modes in each sample. To assess the energies and photon loss rates (*κ*) of modes in each cavity sample, we fit them to Lorentzain lineshapes and extracted the peak positions and widths. Comparisons between the measured and modeled spectra can be found in Figures S4 and S5 of the Supporting Information document. We show the angle-dependent mode energies of the PC and MPC samples in the bottom panels of Figure 3 and compare these values to models of the cavity photon, $E_{c}\left(\theta \right)$ , which can be approximated as [29], [30]

(1) $$E_{c}\left(\theta \right)\approx E_{\text{cutoff}}\sqrt{\frac{1}{1-\frac{\sin^{2}\left(\theta \right)}{n_{\text{eff}}^{2}}}},$$

where *E*_cutoff_ is the energy of the mode at *θ* = 0 and *n*_eff_ is the effective index of refraction of the sample’s intracavity region in the presence of the cavity-embedded two-level systems.

**Figure 4:**
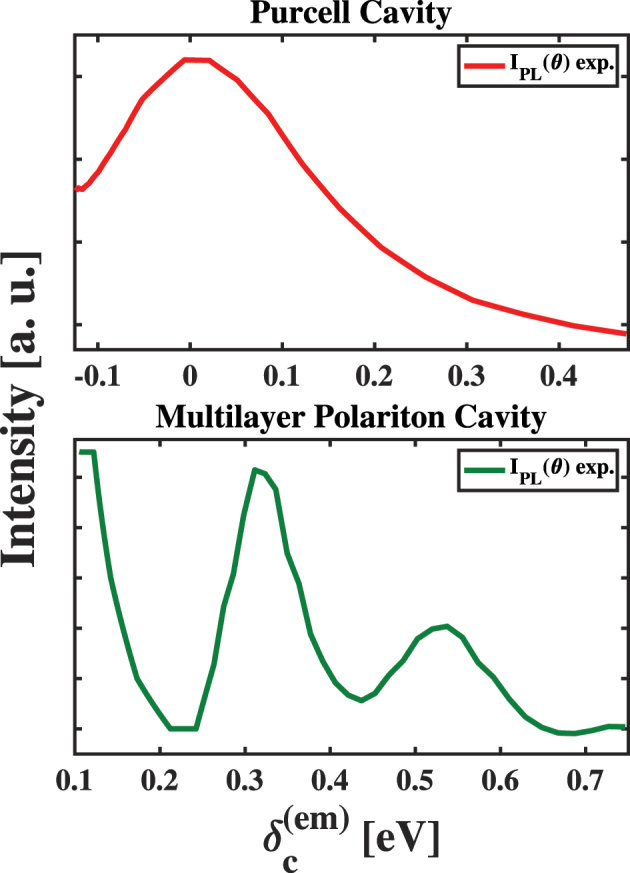
Comparisons of the angle-dependent enhancement of ∼1.92 eV light emission from the Purcell cavity sample (top) and the multilayer polariton cavity (MPC) sample (bottom) following photoexcitation at 3.06 eV (405 nm).

Additionally, the bottom panels of Figure 3 show the angle-dependent light emission energies of the PC and MPC samples following photoexcitation at 3.06 eV (405 nm). Inspection of the trends in the peak energies demonstrates that their values remain almost constant for all the incidence and detection angles used in our experiments. The small dispersion of the emission peak energy in the PC sample near the angle that induces resonance between the ^2^ *Q* exciton fluorescence and cavity photon cannot be observed clearly on the scale shown in the bottom left panel of Figure 3. The transmission spectra of the MPC sample show minimal change as a function of incidence angle in the region near the fluorescence emission of CuTPP, as shown in the top right panel of Figure 4. Based on this fact, we did not consider the dispersion of the cavity transmission in our analysis of the angular dependent PL intensity of the MPC sample.

Our simultaneous plotting of the angularly resolved transmission and emission spectra allows us to assess the effect of the detuning between FP micro-resonator mode and emitters in each sample. By taking the difference between the energies of the proximate cavity mode and light emission peaks for each sample, we can define a detuning $\delta_{c}^{\left(em\right)}$ that determines the enhancement of the radiative relaxation rate caused by the presence of the cavity mode in the weak light–matter coupling limit, as explained below. The panels of Figure 4 show the $\delta_{c}^{\left(em\right)}$ -dependence of the intensity of the 1.9 eV peak in the light emission spectra of the PC and MPC samples. In the case of the PC sample, the top panel of Figure 4 shows that we find a single peak when $\delta_{c}^{\left(em\right)}\;\approx$ 0. In contrast, we find multiple peaks for our MPC sample as we adjust the surface normal of the cavity structure with respect to the incident laser source. We discuss these differences in detail below.

**Figure 5:**
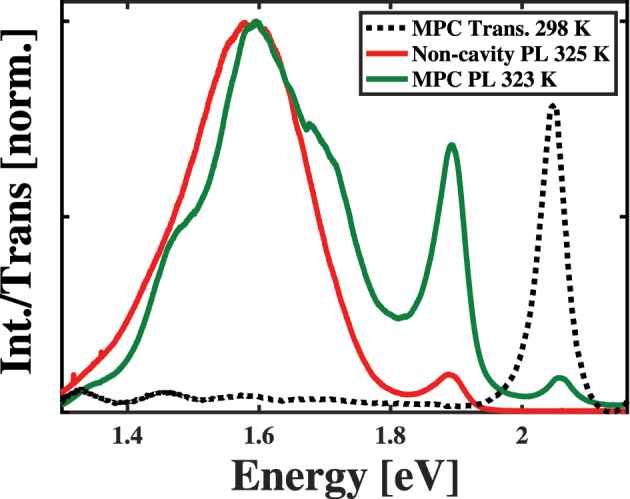
Comparison between the light emission spectra of a non-cavity sample (solid red) and a multilayer polariton cavity sample (solid green) to that of the transmission spectrum of a multilayer polariton cavity sample at normal incidence (dotted black).

Figure 5 shows the transmission spectrum of the MPC sample at normal incidence and the light emission spectra of the MPC and CuTPP-embedded PMMA film samples following 2.33 eV (532 nm) photoexcitation. Inspection of these spectra show that the presence of the lower lying mode in the MPC sample leads to an apparent enhancement of the intensity of the *Q* state light emission relative to a non-cavity sample despite the fact that cavity photons do not overlap energetically with the free space fluorescence. Additionally, we also observe a signal resonant with the cavity mode, which we assign to cavity-enhancement of the 0–0 vibronic contribution to the fluorescence from the CuTPP ^2^ *Q* exciton.

While interesting trends exist between the photonic structures we form and the resulting dependence of their optical spectra on our experimental parameters, practical aspects of our experimental results impede our ability to conclude HT polaritons persist in our MPC samples and cause the behavior of this sample. For example, the top panel of Figure 2 shows that the polaritonic peaks appear with small intensities that complicate concluding that our samples exist in the strong light–matter coupling limit. This facet of our spectra suggests that we need additional methods to support our conclusion of polariton formation, the appearance of the HT polaritons, and any changes the presence of these states should cause in the light emission spectra of our samples. Furthermore, the non-resonant excitation conditions used to make some of our measurements leave the possibility that some other physical mechanism besides HT polariton formation explains our measured spectra. We consider these points in the Discussion section below.

## 3 Discussion

Our experimental results indicate that the presence of cavity polaritons in multimode FP micro-resonators in combination with significant non-Condon vibronic coupling between the ^2^ *B* and ^2^ *Q* excitons of CuTPP leads to complex spectra. To help understand the determinants of our samples’ behavior, we must begin with fundamental models of the intramolecular and light–matter interactions, which take place in both the strong and weak coupling limits.

We recall that the coupling of light and matter within the FP micro-resonator leads to a one-quantum state |*ψ*(*t*)⟩ = $C_{e}\left(t\right){\text{e}}^{-\delta_{c}t/2}|e,0_{c}〉$ + $C_{g}\left(t\right){\text{e}}^{-\delta_{c}t/2}|g,1_{c}〉$ in the interaction picture, where *g* and *e* represent the ground and excited material states, respectively, and 0_*c*_ and 1_*c*_ represent the zero and one photon states, respectively. Solving the time-dependent Schrodinger equation in the weak light–matter coupling limit allows one to show that the rate of light emission from the effective two-level system in a cavity can be written as $\Gamma_{0}+\Gamma_{c}\left(\theta \right)$ [28], where Γ_0_ is the relaxation rate in free space and

(2) $$\Gamma_{c}\left(\theta \right)=\left[\frac{2g^{2}}{\kappa }\right]\frac{1}{1+{\left[2\delta_{c}^{\left(em\right)}\left(\theta \right)/\kappa \right]}^{2}},$$

represents the enhancement to the light emission rate due to the presence of the cavity mode. The terms *g*, *κ*, and $\delta_{c}^{\left(em\right)}\left(\theta \right)$ in Eq. (2) are, respectively, the light–matter coupling strength, photon loss rate, and angle-dependent detuning between the resonator photons and two-level transition frequencies, i.e., $\delta_{c}^{\left(em\right)}\left(\theta \right)$ = $\omega_{c}\left(\theta \right)-\omega_{eg}$ . The material states involved in the light emission transition are separated by the energy *ℏω*_*eg*_. To apply this model to our results, we must first identify those states that participate in the light emission transitions in each of our samples.

The HT Hamiltonian mediates non-Condon vibronic coupling between the *B* and *Q* excitons in porphyrins, which allows the *Q* exciton to borrow transition intensity from the *B* exciton and appear in light absorption and emission spectra [31]. In the case of CuTPP outside the cavity environment, one writes the vibronic quantum state of the ^2^ *Q* exciton to first order as

(3) $$\begin{array}{ll} |Q\left(q\right)〉|m〉 & =|Q\left(0\right)〉|m〉+\underset{m,n}{\sum }\frac{〈Q\left(q\right)|\partial H_{el}/\partial q|B\left(q\right)〉}{E_{B}-E_{Q}}|B\left(q\right)〉\\ & \;\times |n〉〈m|q|n〉\\ & =|Q\left(0\right)〉|m〉+\frac{〈Q\left(q\right)|\partial H_{el}/\partial q|B\left(q\right)〉}{E_{B}-E_{Q}}|B\left(q\right)〉|0〉\\ & \;\times 〈1|q|0〉+\frac{〈Q\left(q\right)|\partial H_{el}/\partial q|B\left(q\right)〉}{E_{B}-E_{Q}}|B\left(q\right)〉|1〉\\ & \;\times 〈0|q|1〉, \end{array}$$

where *q* represents a quantum of the vibrational coordinate responsible for the non-Condon vibronic coupling and *∂H*_*el*_/*∂q* is the HT Hamiltonian. The presence of the factor of *q* in the perturbative ^2^ *Q* exciton wavefunction suggests 0–1 and 1–0 vibronic transitions in light absorption and light emission spectra, respectively, will dominate over 0–0 features. The fourth line of Eq. (3) corresponds to the vibronic correction providing intensity borrowing in the light absorption process to the vibronic state |*Q*⟩|1⟩, while the fifth line of Eq. (3) corresponds to the correction that causes light emission from the state |*Q*⟩|0⟩ to the first vibrational excited level of the CuTPP ground state, which we write as |*G*⟩|1⟩. These assignments of the features in light absorption and light emission have been explained systematically for symmetric metalloporphyrins [32]. In the case of Zn-ligated porphyrins, one can assign this frequency by extracting the energy difference between 0–0 and 0–1 vibronic features in these molecules’ *Q* exciton band, which is about 170 meV (1375 cm^−1^) [32]. In contrast, the relatively higher symmetry of the Cu(II)-ligated porphyrins reduces the intensity of the 0–0 vibronic peak and inhibits researchers’ ability to assign the non-Condon-active vibrational mode directly [33], [34]. Based on this understanding, we understand that the fluorescence spectrum of CuTPP in free space is dominated by the transition between the vibrationless ^2^ *Q* exciton and the first vibrationally excited state of *G*, which takes place near 1.9 eV. The presence of a cavity mode near resonance with this transition should enhance the radiative relaxation rate according to Eq. (2).

When one forms cavity polaritons using the Soret resonance of a metalloporphyrin, we propose that the *Q* excitons of those molecules strongly coupled to the cavity photons borrow transition intensity from the polariton states instead of the bare *B* exciton. In the case of collective strong light–matter coupling, we write the upper polariton (UP) and lower polariton (LP) states as solutions to the Tavis–Cummings model [35], which take the form

(4a) $$|UP〉=c_{ph}^{UP}\left(\delta_{c}\right)|G〉|1_{c}〉+\frac{1}{\sqrt{N}}\overset{N}{\underset{i}{\sum }}c_{ex,i}^{UP}\left(\delta_{c}\right)|B_{i}〉|0_{c}〉,$$

(4b) $$|LP〉=c_{ph}^{LP}\left(\delta_{c}\right)|G〉|1_{c}〉+\frac{1}{\sqrt{N}}\overset{N}{\underset{i}{\sum }}c_{ex,i}^{LP}\left(\delta_{c}\right)|B_{i}〉|0_{c}〉,$$

where $c_{ph}^{i}$ and $c_{ex,i}^{j}$ are the Hopfield coefficients characterizing the composition of the *j*th polariton state [36], which depend on the photon–exciton detuning energy, *δ*_*c*_. Additionally, |*G*⟩ is the ground state of the metalloporphyrin system, and |0_*c*_⟩ and |1_*c*_⟩ represent those states with zero or one photon in the cavity mode. Examining these states, we do not expect the HT Hamiltonian to mediate an interaction between the photonic contributions of the polariton states and the *Q* excitons since ⟨*G*|*∂H*_*el*_/*∂q*|*Q*⟩ ≈ 0. Using this fact, we can expand the *Q* exciton wavefunction in perturbations from the HT Hamiltonian using only the material contributions to Eqs. (4a) and (4b) to find

(5) $$\begin{array}{ll} |Q^{\prime }\left(q\right)〉|m〉 & =|Q\left(0\right)〉|m〉+\frac{1}{\sqrt{N}}\underset{m,n}{\sum }\overset{N}{\underset{i}{\sum }}c_{ex}^{UP}\left(\delta_{c}\right)A_{Q,UP}\\ & \;\times |B_{i}\left(q\right)〉|n〉〈m|q|n〉+\frac{1}{\sqrt{N}}\underset{m,n}{\sum }\overset{N}{\underset{i}{\sum }}c_{ex}^{LP}\\ & \;\times \left(\delta_{c}\right)A_{Q,LP}|B_{i}\left(q\right)〉|n〉〈m|q|n〉, \end{array}$$

where

(6a) $$A_{Q,UP}=\frac{\left\langle Q\left(q\right)|\partial H_{el}/\partial q|B_{i}\left(q\right)\right\rangle }{E_{UP}-E_{Q}},$$

(6b) $$A_{Q,LP}=\frac{\left\langle Q\left(q\right)|\partial H_{el}/\partial q|B_{i}\left(q\right)\right\rangle }{E_{LP}-E_{Q}}.$$

We equate |*Q*′(*q*)⟩ in Eq. (5) with the HT polariton state, |*HT*⟩. The dependence of the Hopfield coefficients and polaritonic energies *E*_*UP*_ and *E*_*LP*_ on *δ*_*c*_ helps explain the minimal dispersion of the energy of |*HT*⟩ found previously [8]. Also previously, matrix diagonalization methods in the presence of static disorder predict that these states should lie nearly 10 meV below those of the bare ^2^ *Q* exciton [8]. The form of Eq. (5) shows that one should expect a $\sqrt{N}$ enhancement when the |*HT*⟩ state participates in radiative relaxation [37], as observed in molecular aggregates [38], [39], [40] and cavity polaritons formed from other organic molecules [25].

In the case of the MPC sample, we propose the vibrationless HT polariton with zero photons $\left(|HT,0〉|0_{c}^{\left(1\right)},0_{c}^{\left(2\right)}〉\right)$ represents the material excitation that interacts with the state of one photon loaded in the lower cavity mode in the presence of a single vibrational excitation on the molecule’s ground state, e.g., $|G,1〉|0_{c}^{\left(1\right)},1_{c}^{\left(2\right)}〉$ . The gap between these theoretical states matches the peaks we find experimentally. The photonic state we expect to participate in radiative relaxation of the HT polariton in the strongly coupled CuTPP-multimode FP micro-resonator resembles the vibrationally dressed cavity photon state, which Spano and Herrera write as [25], [26]

(7) $$\begin{array}{ll} & |\beta ,\tilde{1},0_{c}^{\left(1\right)},1_{c}^{\left(2\right)}〉=\overset{N}{\underset{n}{\sum }}c_{\beta_{n}}|g_{1}0_{1},\ldots .,g_{n}{\tilde{1}}_{n},\ldots .,g_{N}0_{N},0_{c}^{\left(1\right)},1_{c}^{\left(2\right)}〉, \end{array}$$

where *β* represents the permutation quantum number of the wavefunction and the tilde indicates a molecule in its vibrationally excited state, which should have one quantum of excitation to fulfill the photophysics described by the HT Hamiltonian shown in Eqs. (3) and (5). In this case, one might imagine that vibrational excitation shifts the photon energy to produce the detuning $\delta_{c}^{\text{HT}}\left(\theta \right)$ = $\omega_{c}\left(\theta \right)-\omega_{\nu }-\omega_{eg}$ , where *ω*_*ν*_ is the frequency of the molecular vibration that dresses the cavity photon. Based on this proposal from our previous work, $\delta_{c}^{\text{HT}}\left(\theta \right)$ induces an effective resonance condition between the HT polariton and vibrationally dressed cavity photon states, which could lead to the light emission behavior of the MPC sample shown in Figures 3, 4, and 5.

**Figure 6:**
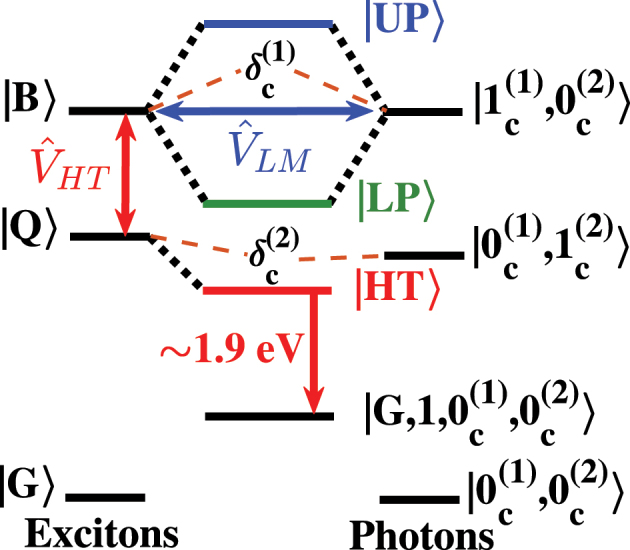
Schematic representation of the energy states and intramolecular interactions of copper(II) tetraphenyl porphyrin (CuTPP) necessary for consideration as molecules of this species couple to photons in the multiple cavity modes of the multilayer polariton cavity sample. We use a downward red arrow to show light emission from the Herzberg–Teller polariton state, |*HT*⟩, to the vibrationally dressed state, $|\beta ,\tilde{\nu },0_{c}^{\left(1\right)},0_{c}^{\left(2\right)}〉$ .

Figure 6 shows the energy states that we propose participate in the photophysics of our MPC sample based on these models. Two facets of this diagram stand out from most studies of cavity polaritons. First, unlike many previous studies on cavity polariton formation using metalloporphyrins, we consider the non-Condon vibronic coupling between these molecules’ spectroscopic ^2^ *B* and ^2^ *Q* states, which we show as ${\hat{V}}_{HT}$ in Figure 6. As explained above, we expect that the combination of ${\hat{V}}_{HT}$ and strong light–matter coupling should lead to the formation of HT polaritons according to Eq. (5), which we denote |*HT*⟩ in Figure 6. Second, the design of our FP micro-resonator enables the formation of two cavity modes separated by one unit of the free spectral range, whose energies lie close to those of transitions to the ^2^ *B* and ^2^ *Q* exciton states of the CuTPP from this molecule’s ground state. In a product basis, we express *n* and *m* excitations in the higher and lower cavity modes, respectively, as |*m* ^(1)^⟩|*n* ^(2)^⟩ = |*m* ^(1)^, *n* ^(2)^⟩. In the case of this multimode FP micro-resonator, we need to define parameters that characterize the light–matter interactions involved in the absorption by the ^2^ *B* exciton and emission from the ^2^ *Q* exciton of CuTPP. We denote the energies of the two resonator modes of interest as *ℏω*_1_ and *ℏω*_2_, which are detuned from the 0–0 vibronic transitions of Soret and Q resonances by values $\delta_{c}^{\left(1\right)}$ and $\delta_{c}^{\left(2\right)}$ , respectively, as denoted in Figure 6 schematically.

**Figure 7:**
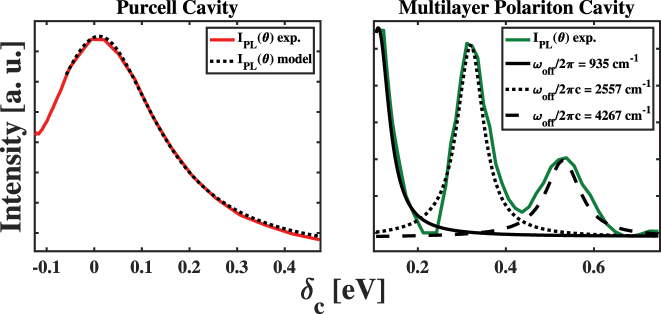
Comparisons of the angle-dependent enhancement of ∼1.92 eV light emission from the Purcell cavity sample (left) and multilayer polariton cavity (MPC) sample (right) to models of cavity enhancements predicted by the conventional and Herrera–Spano theories of weak light–matter coupling, respectively. The peaks in the MPC results appear at detuning values inconsistent with this theory, as explained in the text.

To test the ability of the fundamental models described above to explain our results, we modeled the experimental results shown in the panels of Figure 4 using Eq. (2), which we compare in the panels of Figure 7. In the case of the PC sample, we find that Eq. (2) explains the angle-dependent intensity of the ∼1.9 eV peak when we use *κ* = 300 meV and an appropriate value for *g*, as shown in the left panel of Figure 7. This agreement between our experimental results and theoretical predictions suggests that the PL enhancement in the PC sample stems from the Purcell effect within our FP micro-resonator.

As shown by a comparison of the behavior shown in the panels of Figure 4, we find that the angle-dependent emission intensity of the MPC sample differs significantly from that of the PC sample. To assess the behavior of this sample as a function of $\delta_{c}^{\left(em\right)}$ , we used three separate Lorentzian functions to model the peaks that appear in our results. At the lowest values of $\delta_{c}^{\left(em\right)}$ , we find that the intensity of the light emission peaks and then decreases to a minimum near $\delta_{c}^{\left(em\right)}$ = 200 meV. Based on the model presented above, the peak should stem from the enhancement due to a resonance between the HT polariton and the vibrationally dressed photon state in the lower energy cavity mode. This peak appears near 115 meV, which would correspond to a vibrational frequency of nearly 930 cm^−1^. As stated above, we expect vibrations near 1375 cm^−1^ to participate in the HT coupling, which corresponds to a detuning energy of 170 meV and does not match the value we find experimentally. Furthermore, as we continue to increase the detuning of the cavity photon energy from the ^2^ *Q* state emission peak of film-embedded CuTPP, we find two additional peaks at $\delta_{c}^{\left(em\right)}$ values of 318 meV and 529 meV. These peaks would correspond to vibrations possessing effective frequencies of 2557 cm^−1^ and 4267 cm^−1^, which do not correspond to the energies of known porphyrin molecular vibrations capable of mediating non-Condon vibronic coupling. These facets of our results cause us to consider alternative explanations for the behavior of our MPC sample following photoexcitation at 3.06 eV.

**Figure 8:**
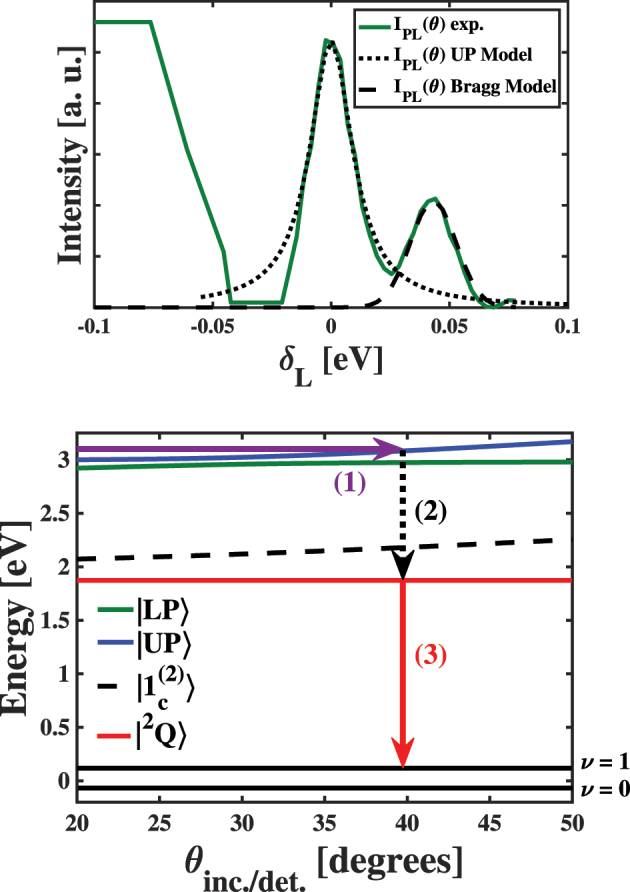
(Top panel) Comparisons between the measured angle-resolved PL excitation spectrum of the MPC sample and model peaks produced with Eq. (7), corresponding to resonant excitation of the UP state and a Bragg sideband. (Bottom panel) Schematic representations of the photophysical processes that we propose drive the appearance of enhanced light emission from the HT polariton to the first vibrationally excited state (*ν* = 1) of CuTPP molecules and enhanced light absorption to the UP state. We show the light emission from the HT polariton state occurring at normal incidence (*θ*_inc_ = 0) as a downward red arrow. Additionally, we represent the enhanced light absorption to the UP state as the value of *θ*_inc_ where the UP state energy matches that of the incident excitation laser, which we show as a horizontal violet line. We assign the peak observed in the top panel at larger *δ*_L_ values as stemming from absorption enhancement due to a Bragg mode of the DBR mirror, which we do not show schematically.

To explain the behavior in the bottom panel of Figure 4, we note the angles at which the incident laser makes with the sample that correspond to these values of $\delta_{c}^{\left(1\right)}\left(\theta \right)$ that may lead to resonances in the absorption of the excitation source by the UP state. To test this hypothesis, we modeled the polariton-enhanced absorption of the 3.06 eV incident laser beam as a function of the detuning $\delta_{L}\left(\theta \right)$ = *E*_*UP*_ − *E*_*L*_ = *E*_*UP*_ − 3.06 eV, which uses the values of *E*_*UP*_ produced by our models of the dispersion curve shown in the bottom panel of Figure 2. Our ability to use these energies to model the angle-resolved light emission spectra of the MPC samples could provide further evidence that our samples reach the strong light–matter coupling limit. For the case of an excitation source in resonance with the polariton state, we propose that the light absorption transition rate becomes enhanced according to the equation

(8) $$\Gamma_{c}\left(\theta \right)=\frac{|c_{ph}{|}^{2}}{{\left[\delta_{L}\left(\theta \right)\right]}^{2}+{\left(\gamma /2\right)}^{2}},$$

where the photonic Hopfield coefficient |*c*_*ph*_|^2^ defines the efficiency with which the incident light enters the polariton state [2], we defined $\delta_{L}\left(\theta \right)$ above, and *γ* represents an effective lifetime of the polariton state. We show our comparison between the measured angle-resolved PL intensity against results of model using Eq. (8) in the top panel of Figure 8. Inspection of this panel shows that the more intense of the two peaks appears when *E*_*UP*_ − *E*_*L*_ ≈ 0, which suggests that these peaks result from the enhancement of light absorption by CuTPP molecules participating in the formation of the UP state. We support this conclusion by noting that CuTPP molecules decoupled from the polariton states absorb very little light at 3.06 eV, as demonstrated by inspection of the linear absorption spectrum in Figure 1. Under these minimal absorption conditions, we expect that only those molecules coupled strongly to the cavity photons can interact resonantly with the incident laser substantially enough to participate in light absorption, localize spatially, and lead to fluorescence of the ^2^ *Q* state. To produce the model peak, we presume *ℏγ* = 22 meV, which is consistent with the linewidth of the UP peak in our transmission measurements. These facets of analysis of the angle-dependent light emission intensity measured experimentally provide further proof that our MPC sample exists within the collective strong light–matter coupling limit.

We find that the second peak appears at $\delta_{L}\left(\theta \right)$ = 43 meV, which we assign as enhancement resulting from overlap of the Bragg mode of the DBR structure with the incident laser energy. Since any cavity mode formed from this Bragg sideband of the main photonic stopband would possess a significantly smaller quality factor than the peak shown as a solid black line in Figure 1, we expect less absorption enhancement, which we observe in the experimental data. Additionally, the relatively reduced quality factor leads to a broader peak when comparing to the feature we observe at $\delta_{L}\left(\theta \right)$ = 0 meV. Furthermore, we need to model this peak as a Gaussian function, which suggests that the enhancement of the light absorption lacks the spatial coherence present in the polariton states. Based on these considerations, we conclude that the additional peaks in the angle-dependent PL intensity spectra stem from cavity-enhanced light absorption, which causes associated increases in the measured PL spectra relative to the constant background light emission. We summarize the photophysical processes we propose account for the dispersive PL intensity of the MPC samples in the bottom panel of Figure 8. First, we vary *θ*_inc_ to tune the energy of the UP state into resonance with the excitation laser, which results in enhanced light absorption by CuTPP participating in cavity polariton formation. Second, the UP polariton localizes onto the *B* exciton of a CuTPP molecule, which relaxes non-radiatively to the lower lying *Q* exciton. Third, the *Q* exciton relaxes radiatively to the first vibrational excited level of the molecular ground state. By measuring the light emission intensity as a function of *θ*_inc_, we can assess where the light absorption peaks and correlate that incidence angle with the detuning between the laser and UP state. We note that this experimental approach depends on the fundamental photophysics of the porphyrin chromophores, which differ significantly from those of other chromophores used conventionally in studies of strong light–matter coupling.

Several theoretical, computational, and experimental studies examining the light emission from polariton samples formed with cyanine dye molecules whose first excited electronic states, *S*_1_, participate in the formation of hybrid light-molecule states [41], [42], [43]. These excited states are displaced significantly from the molecules’ ground state geometries and possess long lifetimes (>10 ps). These properties imply that only wavepacket motion along the *S*_1_ excited state potential energy surfaces (PESs) will act to localize the polariton. There are no electronic states lying at energies below that of *S*_1_ into which non-radiative relaxation would act to localize the polariton on the time scales of its lifetime, which allows researchers to observe complex polarization anisotropies [41] and polariton population trapping [42], [43].

In contrast, the metalloporphyrin molecule we examine in our study, copper(II) tetraphenyl porphyrin (CuTPP), lives for 50 fs in the excited state that participates in polariton formation (*B* exciton) before relaxing to the lower lying *Q* exciton state non-radiatively, as demonstrated in previous studies. The ultrashort lifetime of the *B* exciton in CuTPP implies a near unity quantum yield of forming the *Q* exciton following photoexcitation of bare molecules or cavity polariton states formed from this chromophore. Additionally, this ultrashort lifetime of the *B* exciton limits the region of the *B* state PES ‘probed’ by the nuclear wavepacket projected by the excitation source. Furthermore, the strong aromatic bonding within the porphyrin macrocycle limits the displacement of the *B* state relative to the ground state equilibrium geometry, as we have shown previously [7]. Based on these aspects of the CuTPP structure and dynamics, we expect that these chromophores do not explore regions of the *B* state PES that differ significantly from the equilibrium geometry of the molecule’s ground state.

Given these basic photophysics of CuTPP we expect that cavity polaritons formed from this molecule can localize and relax non-radiatively to the *Q* exciton, which lies nearly 1 eV below the polariton states. These localized molecules can then emit light, which can be controlled by the presence of an additional resonant cavity mode. This behavior differs significantly from those polariton states formed from cyanine dyes unable to relax to lower lying electronic excited states. Therefore, we should not expect that polaritons formed from metalloporphyrins like CuTPP would behave similarly to those hybrid light–matter states comprised of cyanine dyes.

While our angle-resolved light emission measurements reject the hypothesis that vibrations can dress the energies of cavity photons to induce effective resonances that enhance light emission in our samples, the comparison in Figure 5 shows anomalous enhancement of the 0–1 vibronic transition of CuTPP. This enhancement appears odd since the mode of the cavity does not overlap with the spectral signature of the molecule. In most cases where there is no resonance, we should expect that photonic density of states dictated by the cavity will suppress light emission. However, we observe a relative increase in the fluorescence signal. To explain this behavior, we consider the form of Eq. (5). This theoretical result predicts that the HT interaction mechanism in the presence of cavity polariton formation induces a collective state, which we termed |*HT*⟩. Like other collective states, the form of the wavefunction in Eq. (5) suggests that we should observe a $\sqrt{N}$ enhancement in the radiative rate of the HT polariton state relative to *Q* excitons of CuTPP outside of the cavity environment [37]. We propose that this expected collective behavior causes the measured light emission enhancement even when the nearby cavity mode does not meet the polariton–photon resonance condition.

We note that additional multilayer cavity designs would be capable of inducing a resonance between a photonic mode and the *Q* exciton emission of the embedded CuTPP molecules while maintaining a higher energy resonator mode red-detuned from the chromophore’s Soret resonance. While such a sample would allow one to assess Purcell-like enhancements caused by the lower lying sample, the presence of higher energy resonance mode may still affect the photophysics of the cavity embedded molecules in ways that resemble the behavior of the MPC sample with a detuning near −80 meV. As such, spectroscopic results gained using those multilayer samples would not be able to reject the hypothesis that HT polaritons affected their photophysics in the region of the CuTPP *Q* fluorescence as sufficiently as spectra measured using the PC sample we study here.

## 4 Conclusions

In conclusion, we fabricated and characterized two separate sets of cavity samples capable of interacting with the distinct electronic transitions of copper(II) tetraphenyl porphyrin in the weak and strong light–matter coupling limits. By examining changes in the position and intensity of the peak we assign to radiative relaxation from this molecule’s ^2^ *Q* state, we found that a single mode Fabry–Pérot micro-resonator leads to an enhanced light emission process consistent with the trends explained by the Purcell effect previously for completely inorganic systems [44]. In contrast, when strongly coupled to the Soret transition of CuTPP, we find that the lower lying mode of the multimode FP micro-resonator leads to enhanced radiative relaxation near that of the molecule’s ^2^ *Q* state despite being detuned from this light emission peak by over 100 meV. Based on this difference in the angle-dependent behavior of the light emission peaks in each sample, we propose that the states involved in radiative relaxation differ between the two micro-resonators. While we propose that localized CuTPP molecules emit photons into a cavity mode resonant with the light emission transition energy, we are able to reject the hypothesis that Herzberg–Teller polaritons formed in the presence of both strong light–matter and non-Condon vibronic couplings relax radiatively into coherently coupled cavity-vibrational states of these multimode samples. Instead, we find that the form of perturbative wavefunctions for the HT polariton possesses collective features like other hybrid light–matter states and molecular aggregates consistent with enhanced radiative decay. We propose that these collective effects cause enhanced light emission in our multi-layer cavity samples. We delineate this light emission enhancement with other features in the angle-dependent fluorescence spectrum that we argue stem from cavity-enhanced light absorption. These results demonstrate how the behavior of both photons and electrons in complex resonators and molecules, respectively, can be utilized to control the light emission properties of polaritonic platforms towards their application in real-world devices and the care researchers need to take in controlling these properties.

## Supplementary Material

Supplementary Material Details
