# Supplementary material for: Deciphering between enhanced light emission and absorption in multi-mode porphyrin cavity polariton samples
Source: Nanophotonics. 2024 Mar 15;13(14):2695–706. doi: 10.1515/nanoph-2023-0748 (PMC11636455; doi:10.1515/nanoph-2023-0748)
Supplement: Supplementary file 1 — Supplementary Material Details [file j_nanoph-2023-0748_suppl_001.pdf]

Supplemental Material for Deciphering Between Enhanced Light Emission and Absorption in Multi-mode Molecular Cavity Polariton Samples

Elizabeth O. Odewale, Aleksandr G. Avramenko, and Aaron S. Rury[a)]

Materials Structural Dynamics Laboratory, Department of Chemistry,

Wayne State University, Detroit, MI, USA 48202

[a)]Electronic mail: arury@wayne.edu

LIST OF FIGURES

S1 Angularly resolved transmission spectra of the multilayer polariton cavity sample showing the avoided crossing of the cavity photon mode and the CuTPP Soret transition resulting in the appearance of two distinct peaks at θ_{inc} = 29°. At angles below this value we only observe the peak due to transmission through the cavity at energies below the Soret transition of CuTPP. In addition, at angles above 29° we only observe the peak due to transmission through the cavity at energies above the Soret transition of CuTPP, as indicated in the figure. S6

S2 Schematic comparison of the experimental geometry used to assess the dispersive light emission spectra of copper(II) tetraphenyl porphyrin molecules in different cavity samples. S7

S3 Schematic representation of the multilayer polariton cavity sample used to form cavity polaritons from copper (II) tetraphenylporphyrin. The distributed Bragg mirror in this sample was formed from 11 alternating layers of SiO_2 and Si_xN_y were deposited on the silica substrates at a thickness of 174.79 nm.The cavity was designed to support high-Q cavity modes at $\sim$430 nm and $\sim$640 nm for normally incident light, as shown in Fig. 2 of the main manuscript. S8

S4 Schematic representation of the multilayer polariton cavity sample used to form cavity polaritons from copper (II) tetraphenylporphyrin. The distributed Bragg mirror in this sample was formed from 11 alternating layers of SiO_2 and Si_xN_y were deposited on the silica substrates at a thickness of 174.79 nm.The cavity was designed to support high-Q cavity modes at $\sim$430 nm and $\sim$640 nm for normally incident light, as shown in Fig. 2 of the main manuscript. S9

S5 **Top left panel**: transmission spectra of the Purcell cavity sample across different probe incidence angles. **Top row of right panels**: comparisons between the measured transmission spectra of the Purcell Cavity sample to a Lorentzain model explained in the text for incidence angles of 0° (left), 10° (middle), and 20° (right). **Bottom row of right panels**: comparisons between the measured transmission spectra of the Purcell Cavity sample to a Lorentzain model explained in the text for incidence angles of 30° (left), 40° (middle), and 50° (right). S10

S6 **Top left panel**: transmission spectra of the multilayer polariton cavity (MPC) sample across different probe incidence angles. **Top row of right panels**: comparisons between the measured transmission spectra of the MPC sample to a Lorentzain model explained in the text for incidence angles of 20° (left), 22° (middle left), 24° (middle right), and 26° (right). **Bottom row of right panels**: comparisons between the measured transmission spectra of the MPC sample to a Lorentzain model explained in the text for incidence angles of 28° (left), 30° (middle left), 34° (middle right), and 36° (right). S11

S7 **Top two rows of panels**: comparison between the light emission spectra of the Purcell Cavity sample excited by a 3.06 eV laser in the backscattering geometry to the model in Eq. (S2). **Bottom two rows of panels**: comparison between the light emission spectra of the Purcell Cavity sample excited by a 3.06 eV laser in the detection normal geometry to the model in Eq. (S2). S12

S8 **Top left panel**: light emission spectra of the multilayer polariton cavity (MPC) sample following excitation at 3.06 eV and measured in the backscattering geometry for different detection angles. **Top row of right panels**: comparison between the light emission spectra of the MPC sample excited by a 3.06 eV laser in the backscattering geometry to the model in Eq. (S2) for detection angles of 2° (left), 8° (middle left), 14° (middle right), and 20° (right). **Bottom row of right panels**: comparison between the light emission spectra of the MPC sample excited by a 3.06 eV laser in the backscattering geometry to the model in Eq. (S2) for detection angles of 24° (left), 28° (middle left), 32° (middle right), and 35° (right).. . . . . S13

I. METHODS

A. Cavity Fabrication Methods

Non-cavity Sample: Commercially available CuTPP was dissolved in 10 mL PMMA-A4 solution obtained from Kayaku advanced materials. The final solution concentration was approximately 2 mM. The mixture was sonicated for 2 minutes and placed in a water bath at 70 °C for 10 minutes. The sample fabrication was completed at the University of Michigan's Lurie Nanofabrication Facility. Using the E-Beam spinner/Hot plate-21 approximately 400 microliters of the prepared PMMA solution was deposited on a fused silica substrate and spun coated using the static disperse method at 4000 rpm for 45 seconds. The silica substrates were obtained from MTI Corporation. The film was cured at 180 °C for 3 minutes. The spun coated substrates were next secured to the Enerjet evaporator instrument holder with polyimide tape. The vacuum chamber was pumped down to approximately 2 micro-Torr to begin aluminum PVD deposition. A layer of 15 nm of Al was deposited on the film at a rate of 4 Angstroms per second.

Purcell Cavity Sample: Commercially available CuTPP was dissolved in 10 mL PMMA-A4 solution obtained from Kayaku advanced material. The final solution concentration was approximately 1 mM. The mixture was sonicated for 2 minutes and placed in a water bath at 70 °C for 10 minutes. To make the Distributed Bragg Reflectors 1x1 inch fused silica substrates were obtained from MTI corporation. Using the Plasmatherm-790 tool located in the University of Michigan's Lurie Nanofabrication facility 11 alternating layers of SiO_2 and Si_xN_y were deposited on the silica substrates at a thickness of 93.90 nm. Using the E-Beam spinner/Hot plate-21 the CuTPP doped PMMA solution was spun coated unto the Distributed Bragg Reflectors at 2400 rpm to create a Fabry-Perot cavity with a thickness of 220.4 nm. The film was cured at 180 °C for 3 minutes. Samples were then secured to the Enerjet evaporator instrument holder with polyimide tape. The vacuum chamber was pumped down to approximately 2 micro-Torr to begin aluminum PVD deposition. A layer of 13 nm of Al was deposited on the film at a rate of 4 Angstroms per second.

Multilayer Polariton Cavity Sample: Commercially available CuTPP was dissolved in 10 mL PMMA-A4 solution obtained from Kayaku advanced material. The final solution concentration was approximately 1 mM. The mixture was sonicated for 2 minutes and placed in a water bath at 70 °C for 10 minutes. To make the Distributed Bragg Reflectors 1x1 inch fused silica substrates were obtained from MTI corporation. Using the Plasmatherm-790 tool located in the University of

Michigan's Lurie Nanofabrication facility 11 alternating layers of SiO_2 and Si_xN_y were deposited on the silica substrates at a thickness of 174.79 nm. Using the E-Beam spinner/Hot plate-21 the CuTPP doped PMMA solution was spun coated unto the Distributed Bragg Reflectors at 4300 rpm. The film was cured at 180 °C for 3 minutes. The spun coated substrates were next secured to the AE evaporator instrument holder using polyimide tape. The instrument was pumped down to 3 micro-Torr. A layer of approximately 143.7 nm of SiO_2 was deposited at a rate of 3 Angstroms per second. After the deposition of SiO_2 the films were heated to 100 °F for 10 hours and allowed to sit for an additional 2 days to ensure any stresses in the film due to the PVD process were dissipated. Next, a layer of PMMA doped with 30% toluene was spun coated from the 1 mM CuTPP/PMMA solution unto the samples using the static disperse method at 5500 rpm for 45 seconds. The samples were cured at 180 °C for 3 minutes. The samples were then secured to the Enerjet evaporator instrument holder with polyimide tape. The vacuum chamber was pumped down to approximately 2 micro-Torr to begin aluminum PVD deposition. A layer of 13 nm of Al was deposited on the film at a rate of 4 Angstroms per second. This deposition process produced a Fabry-Perot cavity with a final thickness of approximately 431.1 nm.

B. Spectroscopic Methods

To carry out steady-state transmission measurements of the FP micro-resonator samples, we used a fiber-coupled deuterium lamp whose output beam we collimated with free space optics that polarized the incident light fields in the plane of our optical table. We took the resulting beam and focused it onto the cavity samples, collected the transmitted light, and coupled it into fiber-based spectrometer (OceanOptics OceanFX). We measured steady-state transmission spectra at different incident angles so we could estimate the dispersive energies of the cavity mode peaks. In addition, we tested for the anti-crossing behavior of the cavity polariton states in the transmission spectra of those samples we designed to maintain strong light-matter coupling. To more clearly isolate the properties of the polariton peaks in our measured transmission spectra, we fabricated DBR structures without converting them into complete cavity samples. Using these DBR blanks as backgrounds for our steady-state spectra allowed us to reduce significant portions of the baseline on the polariton transmission spectra stemming from the highly dispersive reflectivity of the TM mode of the DBR structure.

We used two different experimental apparatus to understand the dependence of the light emis-

sion spectra of our samples on the exciton-photon detuning energy, which can be controlled by changes to the angle at which the cavity normal makes with respect to the directions of incident and detected beams. For one set of measurements, we used a Horiba XPLoRA PLUS micro-spectrometer affixed with a 10x microscope objective to collect steady-state photoluminescence measurements on all the samples described in this study following excitation at 2.33 eV. We made all the reported measurements using this apparatus in a back-scattering geometry. For another set of measurements, we excited our samples resonantly to either the B states of individual CuTPP molecules or the UP state these molecules form in the PC and MPC samples with output of a 3.06 eV (405 nm) laser. As shown schematically in Figure S2, mirrors (M) steer the output of the 3.06 eV laser to an achromat (ach.) to focus the beam through a spatial filter (SF). The filtered output is collected with a plano-convex lens (PC Lens), attenuated with a neutral density filter (O.D.), and made to be polarized in the plane of the optical table using a half waveplate (λ/2). Following a steering mirror, we used a bi-convex lens (BC Lens) to focus the beam onto the samples through the porthole of parabolic mirror, which then collected the samples' light emission in a back-scattering geometry. After collimating the collected light emission, we focused it with a PC Lens, filtered the reflected laser light using a longpass filter (L-pass Filter), coupled the resulting signal into a 0.5 m spectrograph using a light guide, and measured the spectra with a Si CCD camera cooled to 222 K. We mounted the samples on an assembly of stages that enabled independent vertical translation and rotation of the sample position and orientation while maintaining the same focal spot for the incident and detected beams. This experimental configuration simultaneously varies the angles the excitation and detection beams make with the normal of the sample surface, θ_{inc} and θ_{det}, respectively, and allowed us to measure the light emission spectra of PC and MPC samples across detection angle values of 0° to 60°, as shown in Figure 3 of the main manuscript.

II. SCHEMATICS OF CAVITY STRUCTURES

Figs. S3 and S4 show schematic representations of the structures of the cavity samples we fabricated and characterized spectroscopically in the main manuscript. Each schematic shows both the pertinent layers of the resonator structures and the standing cavity modes we propose dominate the behavior of those samples. For example, we propose only the single, λ/2 standing mode dominates the behavior of the Purcell cavity sample, which we show in Figure S3. In contrast, we propose both the 3λ/2 and λ standing modes of the multi-layer polariton cavity sample

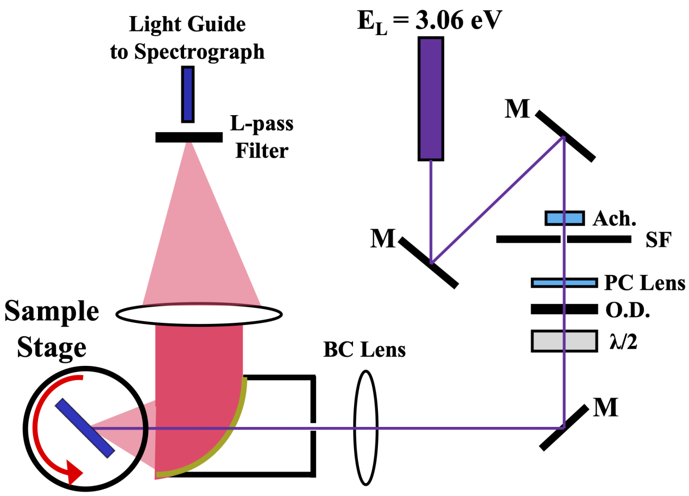

FIG. S1. Schematic comparison of the experimental geometry used to assess the dispersive light emission spectra of copper(II) tetraphenyl porphyrin molecules in different cavity samples.

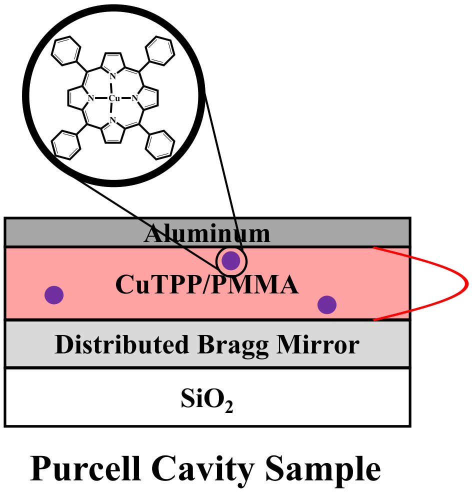

FIG. S2. Schematic representation of the multilayer polariton cavity sample used to form cavity polaritons from copper (II) tetraphenylporphyrin. The distributed Bragg mirror in this sample was formed from 11 alternating layers of SiO_2 and Si_xN_y were deposited on the silica substrates at a thickness of 174.79 nm.The cavity was designed to support high-Q cavity modes at ∼430 nm and ∼640 nm for normally incident light, as shown in Fig. 2 of the main manuscript.

participate in their photophysics, which we show in the resonator schematic of Figure S4. More specifically, the dispersive spectra shown in Fig. S1 above indicate the photons of the 3λ/2 modes of the MPC sample couple strongly to the Soret transition of CuTPP while λ mode couples weakly

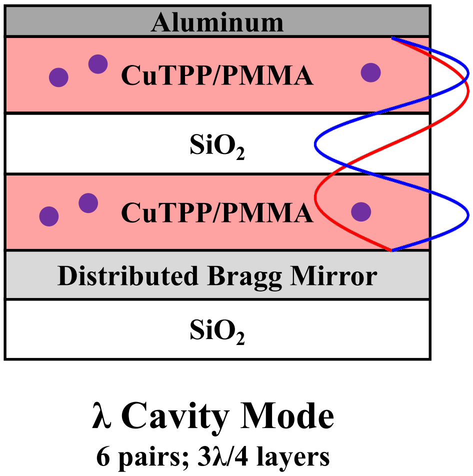

FIG. S3. Schematic representation of the multilayer polariton cavity sample used to form cavity polaritons from copper (II) tetraphenylporphyrin. The distributed Bragg mirror in this sample was formed from 11 alternating layers of SiO_2 and Si_xN_y were deposited on the silica substrates at a thickness of 174.79 nm.The cavity was designed to support high-Q cavity modes at ∼430 nm and ∼640 nm for normally incident light, as shown in Fig. 2 of the main manuscript.

to the light emission from both HT polaritons and uncoupled molecules within the cavity, as seen in the top panel of Figure 1 in the main manuscript.

III. MEASURED AND MODELED RESONATOR TRANSMISSION SPECTRA

To model energetic positions and photon loss rates of the resonator modes in these spectra, we used the following equation,

$$I\left(E,\theta_{\mathrm{inc}}\right)=\frac{I_0\left(\theta_{\mathrm{inc}}\right)}{\left[E_0\left(\theta_{\mathrm{inc}}\right)-E\right]^2+\kappa^2\left(\theta_{\mathrm{inc}}\right)}+\text{baseline}, \tag{S1}$$

where we envision the intensity, I_0, energy, E_0, and mode decay rate, κ, depend on the angle of incidence, θ_{inc}, weakly.

The top left panel of Figure S5 shows the angularly resolved transmission spectra PC sample, which demonstrate our ability to make a well-defined resonator mode near the energy of Q state of CuTPP in this sample. The two rows of panels in Figure S5 compare the PC sample transmission spectra at different incidence angles to models made using Equation S1, which indicate the coherent nature of the photons in the FP micro-resonator modes.

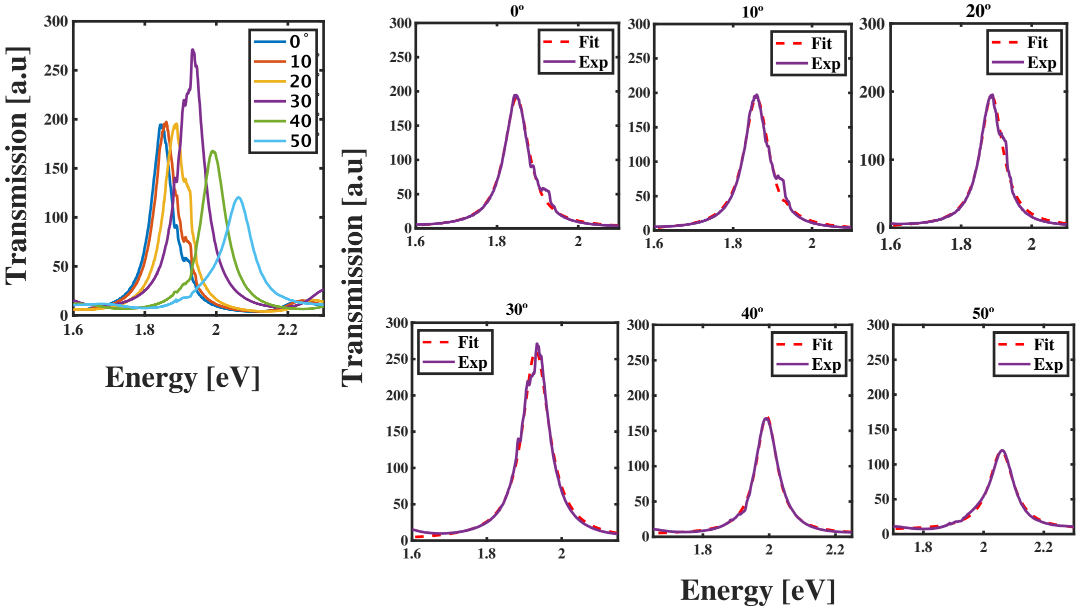

FIG. S4. **Top left panel**: transmission spectra of the Purcell cavity sample across different probe incidence angles. **Top row of right panels**: comparisons between the measured transmission spectra of the Purcell Cavity sample to a Lorentzain model explained in the text for incidence angles of 0° (left), 10° (middle), and 20° (right). **Bottom row of right panels**: comparisons between the measured transmission spectra of the Purcell Cavity sample to a Lorentzain model explained in the text for incidence angles of 30° (left), 40° (middle), and 50° (right).

The top left panel of Figure S6 shows the transmission spectra of the MPC sample in the region near the light emission from the Q state of CuTPP for several incident angles. The two rows of panels in Figure S6 compare the MPC sample transmission spectra at different incidence angles to models made using Equation S1, which indicate the coherent nature of the photons in the FP micro-resonator modes.

IV. MODELING PHOTOLUMINESCENCE SPECTRA

We modeled the spectral region of the angle-dependent PL spectra of PC and MPC samples in the vicinity of CuTPP Q state emission using the following equation,

$$\begin{aligned} I_T(E) &= I_1(\theta_{\text{inc}}) \exp\left(-\left[(E - E_1(\theta_{\text{inc}}))/(2\Delta E_1(\theta_{\text{inc}}))\right]^2\right) \\ &+ I_2(\theta_{\text{inc}}) \exp\left(-\left[(E - E_2(\theta_{\text{inc}}))/(2\Delta E_2(\theta_{\text{inc}}))\right]^2\right), \end{aligned} \tag{S2}$$

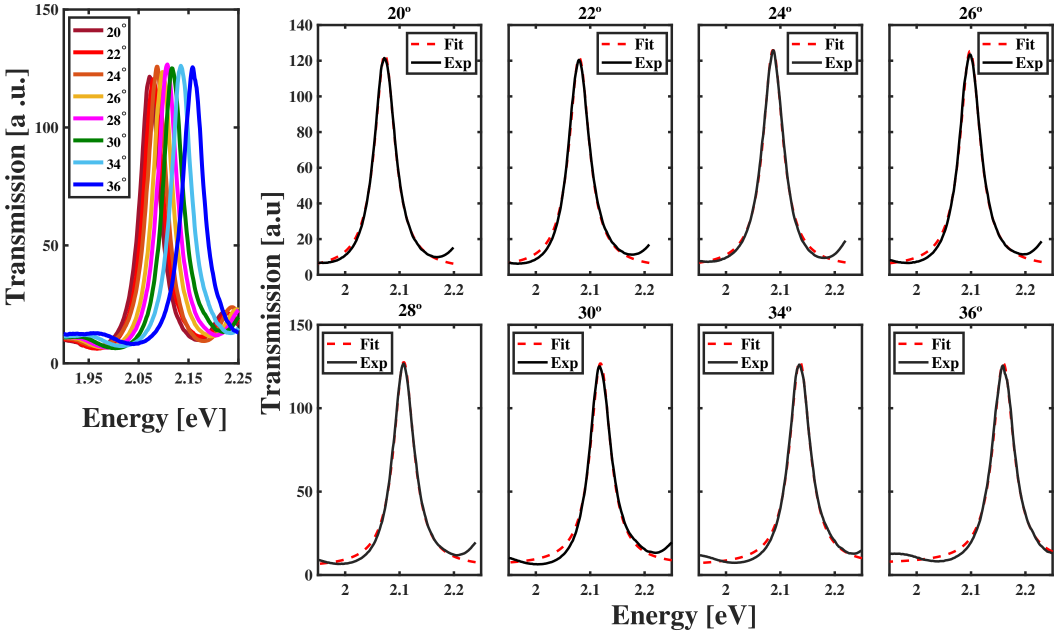

FIG. S5. **Top left panel**: transmission spectra of the multilayer polariton cavity (MPC) sample across different probe incidence angles. **Top row of right panels**: comparisons between the measured transmission spectra of the MPC sample to a Lorentzain model explained in the text for incidence angles of 20° (left), 22° (middle left), 24° (middle right), and 26° (right). **Bottom row of right panels**: comparisons between the measured transmission spectra of the MPC sample to a Lorentzain model explained in the text for incidence angles of 28° (left), 30° (middle left), 34° (middle right), and 36° (right).

where we assign the light emission intensity corresponding to the first and second terms of Eq. (S2) as stemming from the Q states of those CuTPP molecules decoupled from the cavity photons and the resonator-coupled molecules, respectively.

The panels offset to the left side of Figure S7 show the light emission spectra from the PC sample in the different experimental geometries following excitation at 3.06 eV. We show the emission spectra measured using the backscattering geometry in the top row of Figure S7 while those spectra we measure at a normal to incidence direction are shown in the third row. The grid of panels to the right of these offset panels compare the light emission spectra we measure emitted by the PC samples to the model in Eq. (S2) for various detection angle values, which show the ability of the model in Eq. (S2) to explain all the pertinent features of the experimental spectra. We show a similar comparison between experimental and modeled light emission spectra of the MPC sample using Eq. (S2) in the panels Fig. S8 below. These results were measured using the

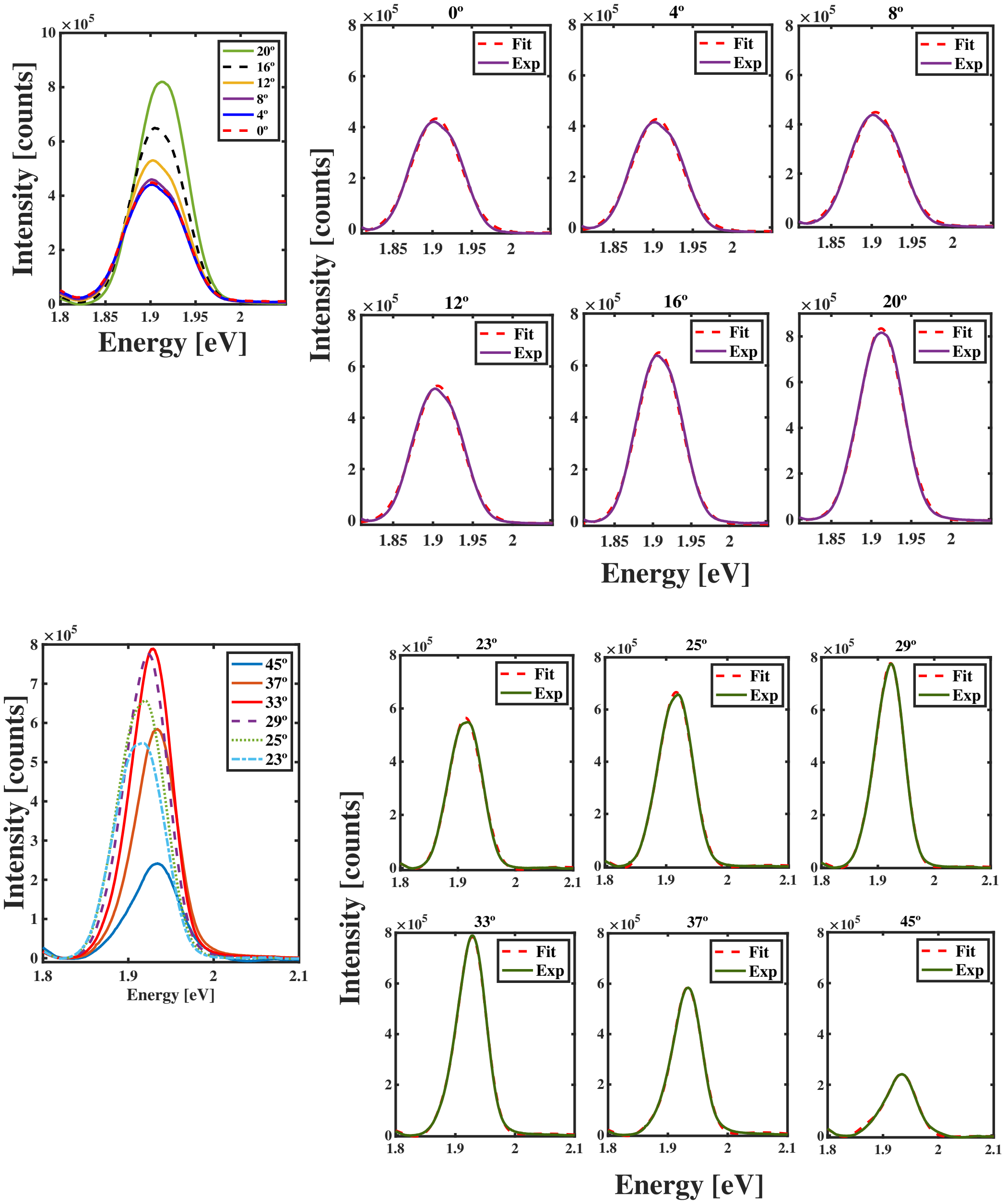

FIG. S6. **Top two rows of panels**: comparison between the light emission spectra of the Purcell Cavity sample excited by a 3.06 eV laser in the backscattering geometry to the model in Eq. (S2). **Bottom two rows of panels**: comparison between the light emission spectra of the Purcell Cavity sample excited by a 3.06 eV laser in the detection normal geometry to the model in Eq. (S2).

backscattering geometry shown in the bottom left panel of Figure S2.

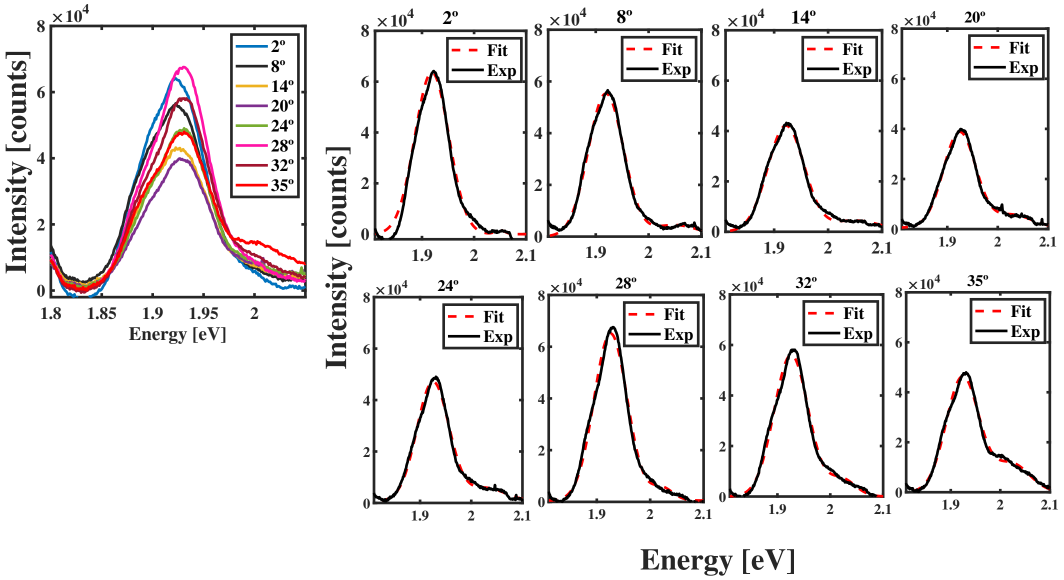

FIG. S7. **Top left panel**: light emission spectra of the multilayer polariton cavity (MPC) sample following excitation at 3.06 eV and measured in the backscattering geometry for different detection angles. **Top row of right panels**: comparison between the light emission spectra of the MPC sample excited by a 3.06 eV laser in the backscattering geometry to the model in Eq. (S2) for detection angles of 2° (left), 8° (middle left), 14° (middle right), and 20° (right). **Bottom row of right panels**: comparison between the light emission spectra of the MPC sample excited by a 3.06 eV laser in the backscattering geometry to the model in Eq. (S2) for detection angles of 24° (left), 28° (middle left), 32° (middle right), and 35° (right)..
